# Passive and Active Exoskeleton Solutions: Sensors, Actuators, Applications, and Recent Trends

**DOI:** 10.3390/s24217095

**Published:** 2024-11-04

**Authors:** D. M. G. Preethichandra, Lasitha Piyathilaka, Jung-Hoon Sul, Umer Izhar, Rohan Samarasinghe, Sanura Dunu Arachchige, Liyanage C. de Silva

**Affiliations:** 1 School of Engineering and Technology, Central Queensland University, Rockhampton, QLD 4701, Australia; l.piyathilaka@cqu.edu.au (L.P.); j.sul@cqu.edu.au (J.-H.S.); s.dunuarachchige@cqu.edu.au (S.D.A.); 2 School of Science, Technology and Engineering (SSTE), University of the Sunshine Coast, Sippy Downs, QLD 4502, Australia; uizhar@usc.edu.au; 3 Department of ICT, Faculty of Technology, University of Colombo, Colombo 00700, Sri Lanka; rohan@ict.cmb.ac.lk; 4 School of Digital Science, Universiti Brunei Darussalam, Gadong 1410, Brunei; liyanage.silva@ubd.edu.bn

**Keywords:** passive exoskeletons, active exoskeletons, sensors, actuators, human augmentation, industrial applications, rehabilitation, wearable technology

## Abstract

Recent advancements in exoskeleton technology, both passive and active, are driven by the need to enhance human capabilities across various industries as well as the need to provide increased safety for the human worker. This review paper examines the sensors, actuators, mechanisms, design, and applications of passive and active exoskeletons, providing an in-depth analysis of various exoskeleton technologies. The main scope of this paper is to examine the recent developments in the exoskeleton developments and their applications in different fields and identify research opportunities in this field. The paper examines the exoskeletons used in various industries as well as research-level prototypes of both active and passive types. Further, it examines the commonly used sensors and actuators with their advantages and disadvantages applicable to different types of exoskeletons. Communication protocols used in different exoskeletons are also discussed with the challenges faced.

## 1. Introduction

The human work force plays a vital role in many critical industries in the modern world. With the growth of the demand for products and the creation of various new occupations, it is evident that labor-intensive routine jobs are losing popularity [1]. Thus, new innovations need to be supporting manual work. Even though fully automated solutions to replace human labor is far from a reality for countries with low or average levels of labor costs [2], solutions that would assist human workers in reducing fatigue and increasing efficiency have an enormous potential. In this regard, many researchers have considered exoskeletal solutions [3]. Wearable exoskeletal implementations mainly focus on reducing the risks associated with manual labor, enabling safe, efficient, and effective task completion [4]. With different implementations of exoskeletons, researchers have developed the two main categories of exoskeletons: passive and active exoskeletons, where the passive exoskeletons are powered by muscle power while the active exoskeletons are powered by external sources such as batteries, pneumatic power, or hydraulics. The passive exoskeletons store human kinetic energy in a form of mechanical energy to later convert it back to kinetic energy again, supplying a assistance to the wearer (energy recycling). Some of the recent developments in powered exoskeletons are welcomed by many industries. Erden and Rainey describe the potential of powered exoskeletons and how the recently developed exoskeletons help manual workers in various industries to perform their work safely and easily [5]. Kim et al. have field tested a whole-body-powered exoskeleton and reported that the user can carry a 50 kg payload during level walking while wearing the powered exoskeleton, with the feeling that they are handling a 6 kg load [6]. This indicates how much support a properly developed powered exoskeleton can provide to a user.

Passive exoskeletons have the inherent advantage of a light weight due to having no power source and actuator requirements, more flexibility, and the ability to work in remote locations for long hours. However, their support to the human worker is limited as the required energy is harvested through human muscles at different times. Active exoskeletal solutions have revolutionized the way human augmentation, rehabilitation, and industrial assistance are perceived. These wearable devices, equipped with sophisticated actuators, hold immense potential in enhancing human capabilities by providing mechanical support, augmenting strength, and facilitating movement. Active exoskeletons employ actuators that are controlled by a computer program based on sensor information during operation. Therefore, they are considered to be more versatile than passive exoskeletons by implementing real-time data through sensors [7]. More physical support can be provided using the advantage of mechanical motors and smart actuation, as compared to passive exoskeletal solutions [8]. The authors have thoroughly reviewed the passive exoskeletons, sensors and actuators used in active exoskeletons, the communication methodologies used in active exoskeletons, and the active exoskeletal solutions for industrial applications in this review.

This article focuses on reviewing the standard sensors and actuators used in exoskeletons, various exoskeleton designs, and the applications of and challenges faced by exoskeletons in different field applications. Recent trends in exoskeleton designs and applications in different fields such as industrial, healthcare, and agriculture are also focused on. 

### Review Methodology

**Figure 1:**
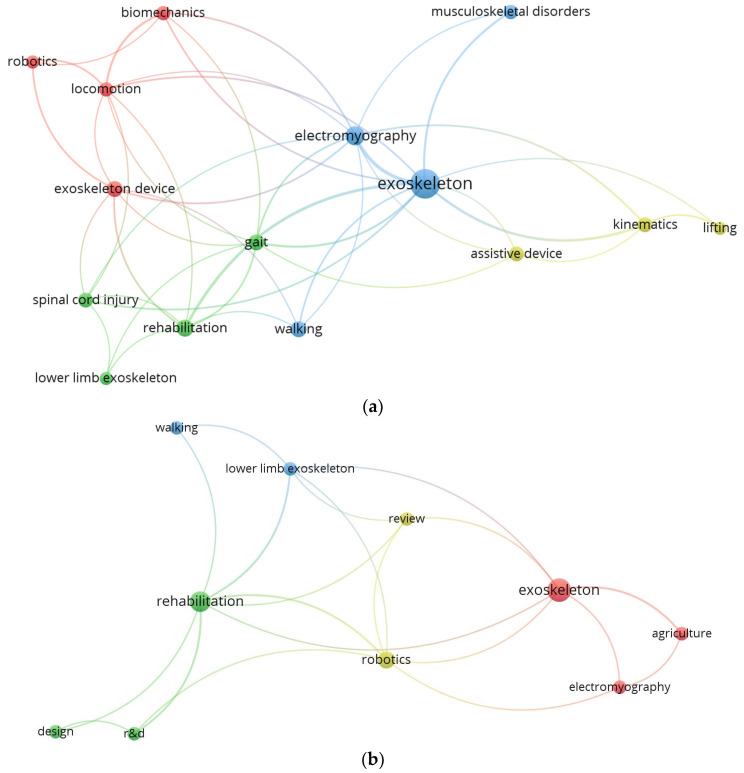
Literature survey bibliographic maps. (**a**) Map for search keyword ‘exoskeleton’ on the lens.org database for 2015–2024. (**b**) Map for keywords in references used in the review.

The literature search related to this review was performed using standard databases such as ScienceDirect, IEEE Explore, Wiley VCH, Taylor and Francis, and Springer. Initially, a keyword search for ‘exoskeleton’ was performed on the online bibliographic database ‘www.lens.org’ for the period of 2015–2024. It was refined for journal articles, book chapters, and conference articles, and the result was 18,728 articles. After analyzing them for keyword commonality with minimum of 4 keywords, this was reduced to 186 articles. Then, VOSviewer bibliographic analyzing software was used to map their commonality as shown in the Figure 1a. The 18,728 articles were further filtered on sub-keywords and the majority of the literature used in this review was identified. Figure 1b shows the bibliographic data map for keyword commonality for the reference list used in this article. The size of each node indicates the number articles using that keyword and the links indicate the connection between the node commonality in both Figure 1a,b.

## 2. Passive Exoskeletal Solutions

Passive exoskeletal solutions have been considered by many researchers for many years to accomplish assistive requirements mentioned in the introduction section. As the name suggests, these exoskeletons do not require an external power to perform the designed assistive task [3]. Alternatively, it can be identified that passive exoskeletons store human kinetic energy in a form of mechanical energy to later convert it back to kinetic energy again, supplying a assistance to the wearer (energy recycling) [4]. Moreover, these inherit real-world advantages such as a longer operational time, simplicity, ease of maintenance, ease of use, and elimination of risks such as electrocution and bulkiness [9,10,11,12]. Also, it can be found in the scientific literature that researchers use these passive exoskeletons as the initiation point for developing much-advanced active exoskeletons. 

The first exoskeleton in the scientific literature appears in 1890 and, without surprise, that was a passive exoskeleton [13,14]. From there onwards, throughout the years, there have been many passive exoskeletal solutions. When analyzing the literature related to passive exoskeletons, it is evident that most of the passive exoskeletons have been designed with the intention of supporting military tasks [4]. However, the declining population and increase in demand had forced researchers to design exoskeletons for various other industries such as agriculture, logistics, manufacturing, retail, etc. Due to that reason, there are multiple commercially available as well as research-prototype exoskeletons that are aimed across various industries, as shown in Table 1.

Before delving into the technical aspects, it is essential to establish a clear understanding of the classification systems used for passive exoskeletons, as these devices can be organized in various ways [3]. A review of the scientific literature reveals that most exoskeletons are designed to provide support to key joints and muscle groups in the human body, including the shoulders, elbows, upper and lower back, hips, fingers, wrists, and knees [3,4,9,10,11,12,13,14,46]. Based on this information, passive exoskeletons can be classified according to the anatomical regions they aim to support. Three primary categories can be distinguished, with further subdivisions within each type.

### 2.1. Upper Limb Exoskeletons

Upper limb exoskeletons are specifically designed to support the user’s upper limbs, as the name implies. These devices may provide assistance to the entire upper limb or target specific joints within the upper limb. As illustrated in Table 1, the majority of commercially available exoskeletons fall within the category of upper limb support. Sub-categories of these exoskeletons include shoulder, elbow, wrist, and finger exoskeletons. A significant portion of upper limb exoskeletons found in the literature employ spring systems or elastic components as the primary passive elements. 

This type of passive exoskeleton has gained popularity due to its ability to support various industries where workers are required to perform tasks with their upper limbs, such as production lines, masonry, carpentry, and fruit or vegetable harvesting [21,26,27]. Passive shoulder exoskeletons, in particular, are frequently designed to assist with overhead tasks by compensating for gravitational forces. In contrast, most lower limb exoskeletons are typically employed in rehabilitation settings, where they assist and guide the user’s movements. 

### 2.2. Lower Limb Exoskeletons 

Passive lower limb exoskeletons are designed to support the wearer’s lower limbs at the hip, knee, and ankle joints. These devices are primarily intended to assist with weight-bearing tasks or to correct and support gait. Depending on the user’s needs, they may be designed to support the entire lower limb or to target specific joints.

However, lower limb exoskeletons are less prevalent in real-world applications compared to upper limb or back exoskeletons, largely due to the unique challenges they face. These exoskeletons must be rigid enough to bear loads while accommodating the complex movements of the hip and ankle joints, which presents significant engineering difficulties. Moreover, the lower limbs have a wide range of motion during various activities, making it challenging to design exoskeletons that allow full freedom of movement.

Although researchers are attempting to address these issues by developing separate modules or exoskeletons for each joint of the lower limb, their performance in load-bearing tasks remains suboptimal. Nevertheless, this modular approach has shown promise in rehabilitation and gait assistance applications [47,48].

### 2.3. Back Exoskeletons 

Back exoskeletons are becoming increasingly popular due to their wide range of practical applications. These passive exoskeletons are designed for use across various industries, including logistics, healthcare, manufacturing, agriculture, and other sectors where workers frequently engage in tasks involving back movements [32,33,34,35,36,37,38].

Most of these exoskeletons provide support to the lower back, enabling individuals to perform lifting, bending, and repetitive twisting tasks more safely, thereby reducing the risk of injury. The required motions for back exoskeletons can be facilitated through traditional mechanical systems that incorporate springs or elastics, as well as through advanced materials, such as newly engineered fabrics. Consequently, this type of passive exoskeleton is attracting increasing interest from researchers worldwide.

In addition to this classification, passive exoskeletons can also be categorized based on the specific tasks they are designed to support. These categories generally include standing, walking, bending, or overhead work [3,49]. 

### 2.4. Design Principles of Passive Exoskeletal Solutions

As the name suggests, passive exoskeletons do not incorporate an assistive element that requires a power source [14]. The approach of designing a passive exoskeleton can be simple or extremely complex, depending on the user’s needs. Nevertheless, the design approach can be described in a few key areas as follows:

#### 2.4.1. Passive Exoskeletal Frame Design

The frame or the mechanical structure of a passive exoskeleton is highly important as it dictates how the exoskeleton would perform. In most of the cases found in the scientific literature, passive exoskeletons consist of a rigid frame that aligns with the wearer’s skeleton and joints and a passive assistive element [14,50]. In most cases, the rigid frame is made out of a lightweight but relatively strong material such as aluminum or carbon fiber [21,42,50,51,52,53].

However, with the development of material technology, there are a handful of exoskeletons that does not have a rigid frame but a “smart” fabric that is specifically designed and fabricated to provide necessary assistance for the wearer [54,55]. These types of exoskeletons have the edge over the traditional passive exoskeletons as they support more free motion/free mobility of the user. Even though these have good potential for mobility, they are limited in providing necessary support for the user in the current context. Fabrication of these types of material can be costly and complex, which ultimately becomes a major hurdle in research and mass production.

#### 2.4.2. Passive Actuator/Element

Even though passive exoskeletons do not incorporate externally powered motion assistive equipment, they rely on various passive elements such as elastic fabric materials [12,15,16,18,19,32,33,35,36,37,38,39,40,41] or spring damper systems [15,16,17,20,21] as their main assistive element. The materials used for passive elements depend on the nature of usage of the element and system requirements. Many exoskeletons with a rigid frame have used springs as their passive assistive element [10,15,17,20,21,22,23,24,25,26,28,29,30,31,42,43,44]. In most cases, these spring base exoskeletons have used the spring as a direct attachment to frame to provide an assistive force to a targeted joint. However, in some special cases, especially in commercially available exoskeletons, developers have been able to develop exoskeleton-specific spring-based mechanical actuators to provide assistive forces [15,16,42,43,44]. The second most-used passive actuators are elastics, as they provide the necessary versatility and flexibility to the exoskeleton [12,15,16,35]. In most cases where elastics are used, similar to springs, they are directly attached to the frame to provide necessary forces and torques. 

There are a few other special types of exoskeletons found in the literature too. The PULE (passive upper limb exoskeleton) [17] is a passive exoskeleton developed based on gas springs. The use of the gas springs allows this exoskeleton to support motions by damping out the forces. On the other hand, VT–Lowe’s exoskeleton [39,40] consists of carbon fiber legs that combine the main two design elements of a passive exoskeleton, the frame and the passive element.

### 2.5. Passive Exoskeleton Maintenance

When considering a real-world scenario, regardless how great the design is, passive exoskeletons are still mechanical systems that require maintenance. Even though none of the exoskeletons found in the literature mention about maintenance, in a mechanical engineering prospective of it, those requirements can be evaluated. Passive exoskeletons with rigid bodies use their passive element quite often; thus, those would eventually wear out. In such an instant, the passive element needs to be replaced. Moreover, it possible to suspect that this might be a major reason for commercial passive exoskeletons to have a replaceable unit as the passive element [11,12,15,17,22,28,29,36,37,38,43,44,56,57]. Even so, passive exoskeletons have more advantages over active exoskeletons, as these passive elements are basically simple mechanical elements. On the contrary, frameless fabric-type passive exoskeletons seem to be replaced once those passive elements are worn out as there is no possible method to repair them. However, if the mass production process can cut costs and make those cheap, replacing these periodically would make more sense.

### 2.6. Passive Exoskeleton Applications

Passive exoskeletons have huge potential in many different areas. In this review, a few of those areas will be presented. One of the main applications of passive exoskeletons is in the military field. This is mainly due to the challenges in the operational environment. As passive exoskeletons do not require additional power sources, they are more suitable to harsh terrain where solders operate. Moreover, these passive exoskeletons act as energy -harvesting devices; thus, the overall efficiency of the solders increases. Also, the military exoskeletons have been designed in a way that they act as armor, protecting the wearer [4,58,59,60].

One other trend that can be seen in the exoskeleton research field is the support of farm work. Specifically, the harsh environments that do not suit for complex electronic equipment such as farms tend to be biased towards passive exoskeletons [17,21,35,41,61]. Most of these exoskeletons, which were introduced for supporting agricultural tasks, seem to be used for gravity compensation and to assist repetitive tasks as that can be identified as major contributions to fatigue and musculoskeletal diseases in agricultural tasks [17,21,62,63,64,65]. 

The manufacturing industry is also another market with high potential for passive exoskeletons. In fact, many of the new passive exoskeletons are aimed towards the manufacturing industry [10,12,17,21,38,39,40,44], since manufacturing processes involve numerous repetitive tasks, overhead tasks, bending tasks, and lifting tasks where workers are at a great risk of work-related musculoskeletal disorders (WMSDs). By introducing a passive exoskeleton, this risk can be greatly reduced [12,24,39]. 

However, rather than observing exoskeletons based on their use cases related to a particular industry, it is much more advantageous to investigate them by the support they can provide. Specifically, in areas such as targeted support area of the human body, it is helpful to investigate their supported motions and suitability to the environment of a particular industry.

### 2.7. Disadvantages

The main disadvantage of a passive exoskeleton is its lack of precision control, which is highly important for a machine that is supposed to be working with a human in harmony. It is evident that this is caused by the use of pure mechanical systems, which inherently have a lack of precision control. Moreover, passive exoskeletons’ range of motions or complex motions capability might be limited due to their constructional constraints and the absence of a closed-loop control system. On top of that, rigid exoskeletons, especially the ones with spring damper systems, might limit the motion of the human while adding additional weight [58].

Despite of all the negative concerns related to passive exoskeletons, it can be observed that these are still highly considered to aid human workers in different industries. The following table depicts exoskeletons that can be found in the literature with their passive elements, target assistive area of human body, and country of origin. When considering the exoskeletons in Table 1, most of them are now commercially available and some are in use in real-world workplaces. Among the passive exoskeletons found in the scientific literature, majority of them are supporting the shoulder, making them suitable for tasks that involve upper limbs. On the other hand, it can be observed that mechanical elements such as springs seems to be used excessively in these commercial exoskeletons.

### 2.8. Recent Trends in Passive Exoskeletons

With the recent developments in engineering, passive exoskeletons seem to be stepping towards a new era specifically with modern fabrication techniques. In the earlier stages of the passive exoskeleton developments, the structure/frame is mainly fabricated with rigid lightweight metals for accomplishing the strength requirement. However, now with the advancements in additive manufacturing, it can be observed that these are being made of new extremely lightweight yet highly rigid materials such as carbon fiber [39,40].

On the other hand, with the new developments in computational technologies, researchers are now capable of predicting the effects of an exoskeleton virtually. OpenSim, a musculo-skeletal simulator that is open source, is a great example of this [66]. OpenSim allows a researcher to attach a designed exoskeleton to a verified human model and observe the muscle activity and the motion of a human subject prior to fabricating the real exoskeleton. Also, it is known that the dynamical analysis of a complex exoskeleton is challenging. However, software such as ADAMS [67] and MATLAB with OpenSim [68] has been able to successfully predict joint torques/forces with minimal error.

According to the review [69,70], it is clear that the scope of passive actuators is ever expanding. Due to the advancement of materials, flexible materials with variable stiffness and damping will allow passive exoskeletons to have more precise control. These developments will allow passive exoskeletons to have more precise control. Moreover, smart fabrics and soft exoskeletons are gaining popularity for their flexible and adaptive nature [69].

## 3. Sensors Used in Active Exoskeletal Solutions

In contrast to passive exoskeletons, active exoskeletons can provide more support through active actuators where the energy comes from a battery bank attached to the exoskeleton for field work or through a wire harness in indoor applications. There are other active exoskeleton types operates on pneumatic power or hydraulic power as well [71]. Very rarely, there are some other sources of power, such as internal combustion engines, which have also been reported [72]. The active exoskeletons with powered artificial muscles or actuators need a tight control of actuation in line with the wearing human’s actions. For this reason, it needs a multitude of information from the human body, including biomedical signals. In addition to that, the exoskeleton needs precise information of each mechanical element’s orientation, position, moving speed, etc. to control the exoskeleton. Recent trends in sensor technology for active exoskeletons focus on improving accuracy, functionality, and integration [71,73]. The integration of multiple sensor types, such as accelerometers, gyroscopes, and magnetometers, into single inertial measurement units (IMUs) provides more precise and reliable motion tracking. Additionally, the incorporation of force and pressure sensors enhances feedback on the forces exerted by and on the user, leading to more accurate support and assistance. Advances in wearable biosensors that monitor physiological parameters, like muscle activity and heart rate, through real-time data collection allow for more personalized and adaptive responses from the exoskeleton [73].

The miniaturization of sensors, driven by advancements in microelectronics, allows for more compact and discreet integration into the exoskeleton’s frame and wearable components [74]. This reduces bulk and weight, improving comfort and overall design. High-precision sensors with improved accuracy and resolution are now able to detect subtle changes in movement and force, contributing to smoother and more natural movement assistance. Additionally, the development of flexible and stretchable sensors, which conform to the user’s body, enhances monitoring accuracy and comfort [75,76].

Innovations in wireless communication and low-power sensors are making exoskeletons more efficient and user-friendly. Wireless sensors eliminate the need for cumbersome wiring, while low-power sensors extend battery life and reduce energy consumption [77]. Emerging trends also include environmental and context-aware sensors that adapt to changes in external conditions, such as terrain and the environment, enhancing the exoskeleton’s versatility and performance in various settings [78]. These advancements collectively aim to improve the functionality and user experience of active exoskeletons, expanding their applications and benefits.

### 3.1. Angle Sensors and Encoders

Angular sensors have been developed measuring different physical quantities varying with the variation of angle. This includes capacitive, inductive, Hall effect-based, and optical sensors [79]. Wu et al. have used COMSOL Multiphysics to simulate novel capacitive angular sensor design and achieved a minimum rotational angular step value of 0.005° with a minimum angle difference of 0.0018° [80].

#### 3.1.1. Capacitive Angle Sensors

**Figure 2:**
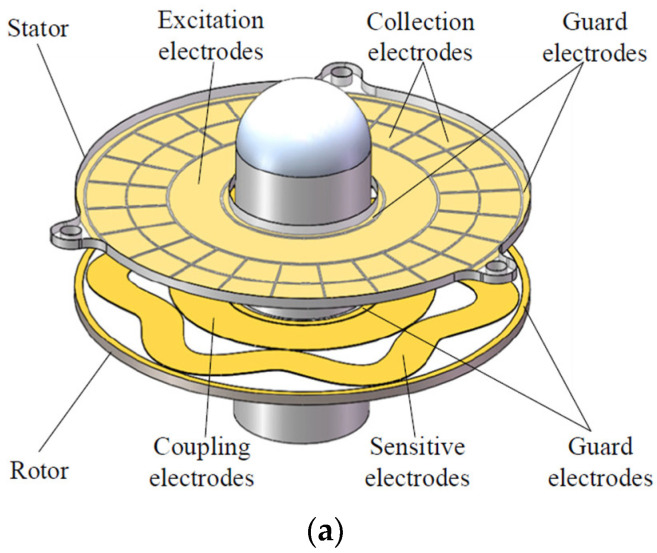

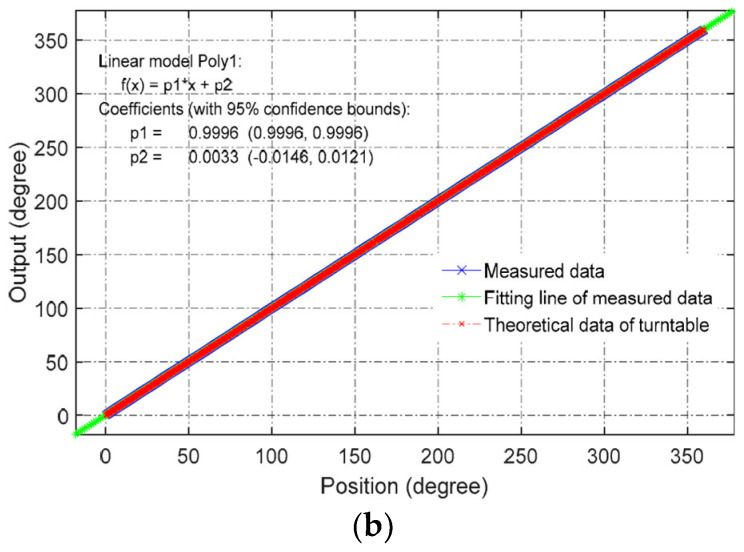
(**a**) Parallel disc capacitive sensor structure and (**b**) experimental output of the sensor [82]. (Reproduced from MDPI Sensors under CC By 4.0 license).

Most of the capacitive angle sensors are made with circular printed circuit board (PCB) structures where one of the two parallel PCBs has radial segments and the other one will have different geometries. George et al. have reported a linear variable differential capacitive transducer (LVDCT) with multiple circular discs producing a highly linear output with less than 0.1% error [81]. Hou et al. produced a different circular capacitive plate sensor as shown in Figure 2, where the linearity of the output and the nonlinearity error are convincing [82]. They have grouped the collection electrodes into four groups and capacitances formed by these grouped electrodes and sensitive electrodes are connected to a specially designed interface circuitry in order to obtain a linear output against the absolute angular rotation. Hou et al. have improved their previous sensor by introducing a micro-fabricating technology and a resolver chip into single module exhibiting a 0.0012° accuracy [83]. Pu et al. have developed an absolute angular position sensor using vernier capacitive arrays [84]. The reported accuracy is within ±2” over 360° measurement range. There are capacitive angle sensors that can be used for exoskeletons to monitor finger and other joint angles. Goto et al. have reported a capacitive bending angle sensor using double layers of conductive elastomers [85]. The accuracy was reported as changing and the root mean square error (RSME) ranged from 4.7° to 7.0°. Therefore, it may not be an ideal candidate for exoskeletal joint angle measurement where the accuracy of the joint angles is crucial. Several other designs of rotary disc-type angle sensors and encoders are reported in the recent literature [86,87,88]. Crea et al. presented a robotic hip exoskeleton with non-contact capacitive sensors [89]. They have reported that the exoskeleton assisted subject had a metabolic expenditure of 3.2% ±1.1 less than that of the unassisted subjects when tested.

#### 3.1.2. Inductive Angle Sensors

Inductive angle sensors have been widely used in industrial environments and they are potential candidates for exoskeletons. There are few different types of inductive angle sensors such as variable mutual inductance-based angle sensors, variable mutual reluctance-based angle sensors, planar coil-based inductive angle sensors, and eddy current-based angle sensors [79]. Anandan et al. reported a planar coil based angular sensor with a resolution of 0.15° with rms error of 0.58% [90]. They have used the linear variable differential transformer (LVDT) model and, due to the geometric shape selected, the linearities in all four quadratures have very similar outputs. External electronic interfacing was designed to make it work for 0° to 360°. Sun et al. developed an inductive displacement sensor with a spur gear wheel and a magnetic probe [91]. They achieved an accuracy of ±0.0012°, which is more than enough for an exoskeleton system to accurately mimic the human actions. A very similar angular displacement sensor was developed by Wu et al., but their excitation and signal processing scheme were different [92]. Tang et al. have developed an angular displacement sensor with a planar coil and a contrate gear wheel where they reported an accuracy of ±12 arcsec over 360° [93]. Tavassolian et al. have developed a fabric-based soft inductive sensor for wearable hip joint angle measurement during running events [94] and this may be a suitable sensor for exoskeleton development.

#### 3.1.3. Hall-Effect Angle Sensors

Hall-effect sensors are commonly used in permanent magnet synchronous motors and brushless DC motors to obtain their relative angular movements and speed. They are widely used in various industrial applications as well. Anoop and George have presented a variable reluctance based angular sensor with a less than 1% error [95]. Palacín and Martínez have developed a low-cost magnetic rotary encoder by compensating for magnet and Hall-sensor misalignments [96]. There are many other Hall-effect based angular sensors developed by different research groups [97,98,99] and all of them are good candidates for exoskeleton joint angle measurements.

### 3.2. Accelerometer Sensors

Accelerometer is a device that outputs a signal due to acceleration in one direction. There are two-axis and three-axis accelerometers, where you obtain the acceleration information on up to three orthogonal (X-Y-Z) directions. They can be categorized as piezoelectric, piezoresistive, and capacitive accelerometers. The piezoelectric accelerometers produce a voltage signal when they face a sudden change in velocity, and they are in demand for measuring shocks and vibrations in industrial applications. Piezoresistive accelerometers display a variation in the resistance when they experience a change in velocity, but they are less sensitive compared to piezoelectric counterparts. Therefore, they are not suitable for low frequency applications and commonly used in high-amplitude high-frequency applications such as vehicle crash-testing measurement systems and weapon-testing systems. The capacitive type accelerometers have two capacitive plates and a diaphragm, where the diaphragm moves in response to an acceleration causing a change in the capacitance. Micro electro-mechanical system (MEMS)-based capacitive accelerometers are commonly used in smart phones and handheld devices today. The multi-axis accelerometers either have multiple single-axis accelerometers mounted perpendicular to each other in the assembly or multiple single-axis accelerometers micromachined on the same substrate on respective orthogonal axes of operation.

**Figure 3:**
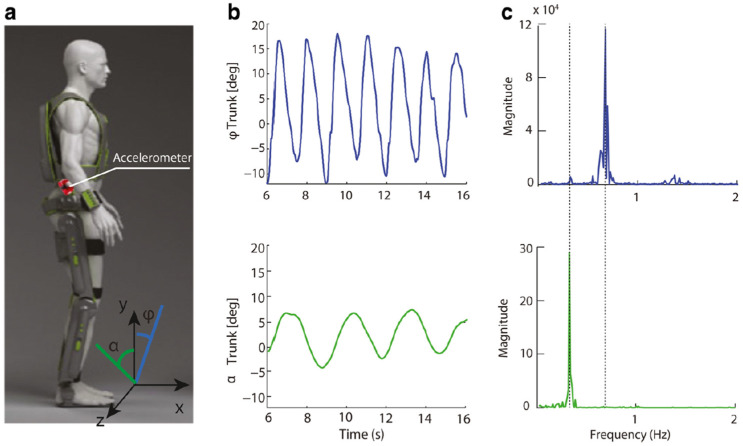
The accelerometer added to ReWalk™ system. (**a**) Schematic of the system. (**b**) Trunk angle in x-y plane (blue line) and in y-z plane (green line). (**c**) The power spectral density of the trunk angles [105]. (Reproduced from Journal of NeuroEngineering and Rehabilitation under CC by 4.0 license).

Accelerometer data from a smart watch has been used by Sharma et al. with their newly developed multi-view data fusion algorithm to recognize the human activity [100]. They have applied their new data fusion algorithm to the harAGE dataset [101] and reported achieving a 6.01% improvement in recognizing human activity. Lazzarone et al. reported improving the efficiency of active back-support exoskeleton system for heavy lifting in the industry [102]. Their new system has reduced the erector spine muscle activity by 34%. An accelerometer-based instrumented crutches for assisted gait exoskeleton users was reported in [103]. They have used a wearable powered exoskeleton Rewalk™ (from Argo Medical Technologies Ltd., Yokneam Illit, Israel) for this study, worn by a male human subject with motor complete SCI (spinal cord injury). They reported that the usage of accelerometer-equipped crutches reduced the support provided by the trainer to the patient by a large factor, up to 40% of the patient’s body weight. Cortese et al. developed a robotic exoskeletal hand for training the hand of a patient [104]. In the system developed, a sensor glove (Acceleglove by AnthroTonix, Silver Spring, MD, USA) equipped with six three-axis accelerometers is worn by the trainer, and the exoskeletal robotic hand attached to the patient’s hand follows the trainer’s hand, controlled by the accelerometer signals gathered. Lonini et al. have attached a tri-axial accelerometer on the right flank of the ReWalk™ exoskeletal system to gather additional data from the system [105]. This accelerometer provides additional data, as shown in Figure 3. They have used different mathematical models to examine the data and found that this method with additional accelerometer data provides more opportunity for the patient to be trained better, proven by three out of four patients who were different from the experts.

### 3.3. Force and Torque Sensors

Force and torque sensors are fundamental in active exoskeleton systems, facilitating precise control and seamless interaction between the user and the device. These sensors are responsible for measuring the mechanical loads applied to the exoskeleton, thereby generating critical data that informs the system’s control algorithms. The accurate data these sensors provide is essential for ensuring that the exoskeleton delivers the appropriate level of assistance, maintains safety, and adapts effectively to the user’s movements and intentions [106,107,108].

Ensuring user safety is a primary concern in the design and operation of exoskeletons. The continuous monitoring of forces and torques by integrated sensors allows the system to detect abnormal conditions, such as excessive force or unexpected torque, which could indicate potential risks or hazardous situations. Upon detecting such anomalies, the exoskeleton can promptly adjust its support or halt operations to prevent injuries, thereby providing a safer interaction between the device and the user [109,110,111].

#### 3.3.1. Strain Gauges

Strain gauges operate on the principle of measuring the deformation (strain) of an object. When an object deforms under the application of force, the electrical resistance of the strain gauge changes. This change in resistance can be accurately measured and correlated to the amount of force applied. The precise measurement of this deformation allows for the detection of even minimal changes in force, making strain gauges highly sensitive and reliable for various applications [111].

In the context of active exoskeletons, strain gauges are commonly utilized in load cells [112] and torque sensors. They are strategically placed at critical points on the exoskeleton to measure the forces exerted by the user. These critical points include joints such as the knees, elbows, and hips, where significant forces and torques are generated during movement [113]. For instance, at the knee joint, strain gauges can measure the forces exerted during walking or lifting, providing data that helps the exoskeleton assist in these actions effectively [114]. Similarly, at the elbow joint, they can monitor the forces involved in tasks requiring arm movement and lifting [115,116].

Strain gauges are also placed along the length of the exoskeleton’s limbs, such as the thighs and forearms, to measure the distribution of forces along these segments [114,115]. This placement helps in understanding how forces are transmitted through the body and the exoskeleton, ensuring that assistance is provided in a manner that mimics natural movement patterns. Additionally, strain gauges can be integrated into the back support structure of the exoskeleton to monitor the forces exerted on the spine, which is crucial for tasks involving lifting or carrying heavy loads [116]. By providing real-time data on force application at these various points, strain gauges contribute significantly to the control, adaptability, and safety of exoskeletons.

#### 3.3.2. Torque Sensors

Torque sensors are essential components in the design and functionality of active exoskeletons, measuring the rotational force, or torque, around an axis [117,118]. These sensors often employ strain gauges or piezoelectric elements to detect the twist or rotational displacement in a shaft or other rotating components. The data obtained from torque sensors are crucial for understanding the forces involved in various movements, enabling the precise control and stability of the exoskeleton.

In the design of exoskeletons, torque sensors are primarily utilized in the joints, where significant rotational forces are generated during movement. For instance, at the knee joint, torque sensors measure the rotational forces exerted during activities such as walking, squatting, or climbing stairs [118,119]. By accurately detecting these torques, the exoskeleton can provide the necessary support and assistance, ensuring smooth and natural movement. This is particularly important in rehabilitation exoskeletons, where controlled and precise assistance is required to aid in the recovery of patients with mobility impairments.

Similarly, torque sensors are used in the elbow joint to monitor and control the rotational forces involved in arm movements [120]. Tasks such as lifting objects, reaching out, or performing repetitive motions generate significant torque at the elbow. The data from the torque sensors allows the exoskeleton to adapt to these forces, providing the appropriate level of assistance and enhancing the user’s strength and endurance. This application is vital in industrial exoskeletons, where workers perform repetitive or strenuous tasks that can lead to fatigue or injury.

Torque sensors are also integrated into the hip joints of exoskeletons, where they measure the rotational forces during walking, running, or lifting [121]. The hip joint experiences complex rotational movements, and accurate torque measurement is crucial for maintaining balance and stability. By monitoring these forces, the exoskeleton can adjust its support dynamically, helping users maintain proper posture and prevent falls. This is especially beneficial for elderly users or those with conditions that affect their balance and coordination.

### 3.4. EMG Sensors

Electromyography (EMG) sensors play a crucial role in the functionality of active exoskeletons, providing a direct interface between the user’s neuromuscular system and the robotic device [122,123]. EMG sensors measure the electrical activity produced by skeletal muscles, offering insights into muscle activation patterns, which can be used to control and fine-tune the exoskeleton’s movements. The integration of EMG sensors into active exoskeletons enhances the precision, responsiveness, and adaptability of these devices [123,124].

#### 3.4.1. Working Principle

EMG sensors function by detecting the electrical potentials produced by muscle cells when they are electrically or neurologically activated. This detection process involves the use of electrodes, which can be either surface electrodes or intramuscular electrodes. Surface electrodes, commonly used in exoskeleton applications, are placed on the skin over the target muscles. These electrodes are typically composed of conductive materials such as silver/silver chloride (Ag/AgCl) and are designed to pick up the small voltage changes that occur when the underlying muscles contract [124,125].

Once the EMG sensors detect the myoelectric signals, these signals, which are typically in the microvolt (µV) range, need to be amplified to be usable. Amplification is achieved through the use of differential amplifiers that increase the signal strength while minimizing common-mode noise. After amplification, the signals undergo a filtering process to remove noise and artifacts. This filtering typically involves the use of bandpass filters, which allow frequencies within a specific range (usually between 10 Hz and 500 Hz) to pass through while attenuating frequencies outside this range. This range is chosen because it encompasses the typical frequency spectrum of myoelectric signals.

The filtered EMG signals are then processed to extract relevant features that represent the user’s muscle activity. Commonly extracted features include the root mean square (RMS), mean absolute value (MAV), and zero crossings. These features are used to quantify the level of muscle activation and are essential for interpreting the user’s movement intentions. Advanced signal processing techniques, such as wavelet transforms and neural network algorithms, may also be employed to enhance the accuracy and reliability of the extracted features.

The extracted features from the EMG signals are then used to control the exoskeleton [19]. This control process involves interpreting the muscle activation patterns to determine the user’s intended movements. For instance, an increase in the RMS value of the EMG signal from the biceps muscle might indicate that the user intends to flex the elbow. The control system of the exoskeleton uses this information to activate the appropriate actuators, thereby assisting the user in performing the desired movement. This process requires sophisticated algorithms capable of real-time processing and adaptive control to ensure that the exoskeleton’s actions are synchronized with the user’s intentions [123].

#### 3.4.2. Applications

Electromyography (EMG) sensors are instrumental in determining user intent by analyzing muscle activation patterns. When a user intends to perform a movement, such as lifting an arm or taking a step, the associated muscles produce specific EMG signals. These signals are detected by the sensors, which then relay the information to the exoskeleton’s control system [126]. By interpreting these signals, the exoskeleton can assist with the movement, ensuring a seamless and intuitive interaction between the user and the device. This application is particularly beneficial in enhancing the user’s physical capabilities, allowing for more natural and fluid movements.

In the context of rehabilitation, EMG sensors play a crucial role in monitoring muscle activity to assess the progress of patients recovering from injuries or surgeries. The data collected from these sensors provide valuable insights into muscle function and activation patterns, which can be used to guide therapists in adjusting therapy programs. This continuous monitoring enables the tracking of improvements in muscle strength and coordination over time. By providing targeted assistance based on real-time EMG feedback, exoskeletons can facilitate more effective rehabilitation, helping patients regain their mobility and independence more efficiently [122,127].

EMG sensors also enable adaptive control strategies in exoskeletons, allowing the device to modulate its assistance based on real-time assessments of muscle fatigue or effort levels [126]. For instance, during prolonged use, if the sensors detect signs of muscle fatigue, the exoskeleton can increase its support to reduce the load on the user. This adaptive capability enhances the endurance and comfort of the user, making the exoskeleton more effective in various settings, such as industrial work or long-term rehabilitation [128]. By continuously adjusting the level of assistance, the exoskeleton ensures optimal performance and user satisfaction.

#### 3.4.3. Challenges

One significant challenge in the use of electromyography (EMG) sensors in active exoskeletons is signal variability. EMG signals can vary significantly due to factors such as electrode placement, skin conductivity, and muscle fatigue [129]. These variations necessitate the development and implementation of robust signal processing algorithms capable of compensating for such inconsistencies to ensure accurate and reliable interpretation of muscle activity [130].

Another challenge is the susceptibility of EMG signals to noise and artifacts [131]. These signals are prone to interference from external sources and movements, which can obscure the true muscle activity signals. This issue requires the application of effective filtering techniques to isolate the relevant EMG signals from noise, thereby enhancing the signal-to-noise ratio and ensuring the fidelity of the data used for controlling the exoskeleton.

Furthermore, the effective use of EMG sensors in exoskeletons often requires user training. Users must learn to generate consistent EMG signals to achieve optimal control over the exoskeleton [132]. This training process is crucial for enabling users to effectively communicate their movement intentions to the device, thereby enhancing the overall performance and user experience of the exoskeleton.

### 3.5. Comparative Analysis of Sensor Technologies

The sensor types discussed in the previous section play a critical role in facilitating effective interaction between exoskeletons and their users. These sensors provide essential data for motion tracking, force feedback, and user intent recognition. A comprehensive understanding of the advantages and disadvantages associated with each sensor type is vital for optimizing the design and performance of exoskeleton systems [71].

Inertial measurement units (IMUs) serve as fundamental components of many exoskeletons, recognized for their capacity to capture high-frequency data, which is critical for real-time motion tracking. Their compact size and affordability contribute to their popularity among developers. However, IMUs encounter significant challenges, particularly, a susceptibility to drift over time, which can result in inaccuracies in motion data [71]. To maintain precision, these sensors require regular calibration, and their effectiveness may be compromised during complex movements that necessitate multi-directional tracking.

Force sensors provide valuable insights by directly measuring the forces exerted during user interaction with the exoskeleton. This capability allows for real-time feedback on user effort and plays a crucial role in enhancing control algorithms that govern the device’s responses. Despite these advantages, force sensors present certain drawbacks. Their range and sensitivity can be limited, particularly in dynamic scenarios, and they may experience wear and tear under heavy loads, necessitating regular maintenance and replacement [133].

Torque sensors are particularly important in exoskeletons due to their ability to accurately measure rotational forces, enabling the precise control of joint movements. This capability is essential for executing complex tasks that require fine motor skills and coordination. However, the integration of torque sensors can be challenging because they are typically more expensive than other sensor types. Installation may also be complex, requiring careful alignment to ensure accurate readings, and these sensors may exhibit limited sensitivity at lower torque levels, which can diminish their effectiveness in certain applications.

Pressure sensors excel in measuring contact forces, facilitating the detection of user engagement with the exoskeleton. Their small and flexible designs enable easy integration into various components of the device. Nevertheless, pressure sensors have inherent limitations. They often require careful calibration to maintain accuracy and are generally restricted to static or quasi-static measurements. This limitation can impact their effectiveness in dynamic environments, where rapid changes in pressure may not be accurately captured. Furthermore, external factors such as temperature variations can influence their performance, potentially leading to inconsistent readings.

Angle sensors provide precise angular position data, which is critical for measuring joint angles and facilitating motion control in exoskeletons. The ability to obtain accurate angular measurements enhances the device’s responsiveness and adaptability to the user’s movements. However, angle sensors are not without challenges. They can be sensitive to mechanical wear over time, leading to potential inaccuracies if not properly maintained. Calibration may also be necessary to ensure accurate readings, and certain sensor designs may have a limited range, restricting their applicability in specific configurations.

EMG sensors represent a cutting-edge technology that directly measures muscle activation, enabling intuitive control of the exoskeleton by mimicking the user’s natural movements. This capability allows for seamless integration of the device into the user’s physical activities, enhancing usability and effectiveness. However, EMG sensors face a distinct set of challenges. They can be adversely affected by noise and crosstalk from nearby muscles, which may compromise the accuracy of the readings. The proper placement of these sensors is critical for achieving reliable measurements, and user-specific calibration is often necessary to accommodate individual differences in muscle physiology [130].

Comprehending the advantages and limitations of these various sensor technologies is essential for optimizing the design and performance of active exoskeleton systems. As the field continues to evolve, ongoing research and development will be necessary to address these challenges and enhance the functionality, comfort, and effectiveness of exoskeletons across diverse applications

## 4. Actuators Used in Active Exoskeletal Solutions

Active, also known as powered, exoskeletons can provide more physical support than passive exoskeletons by using the advantage of actuators, such as electric, pneumatic, hydraulic, and smart actuators [8]. Actuators, serving as the core components of active exoskeletons, are responsible for converting energy into mechanical motion, thereby enabling a wide range of applications across various domains. However, these powered wearable devices are often bulkier in design and typically heavier compared to passive exoskeletons, thereby potentially hampering the net gain for the wearer and decreasing their intended performance [134]. Therefore, the selection of actuators for an active exoskeleton must be made through a systematic and logical approach. The choice of actuators is dependent on critical parameters, such as power/mass ratio, power volume ratio, stress, strain, steady-state efficiency, power consumption, bandwidth, auxiliary transmission system, auxiliary power supply equipment, and ease control procedures [135]. Recent advancements in actuator technology for active exoskeletons highlight significant improvements in performance, versatility, and integration. Traditionally, actuators in exoskeletons were primarily based on electric motors, hydraulic systems, or pneumatic mechanisms. However, there has been a notable shift towards the development of soft actuators, such as artificial muscles [136] and electroactive polymers [137], which offer a more flexible and responsive approach to movement assistance. These soft actuators can mimic natural muscle contractions more closely, providing smoother and more adaptive support that enhances the overall user experience and reduces mechanical rigidity.

Additionally, there is increasing interest in hybrid actuator systems that combine multiple actuation technologies to leverage their respective advantages. For instance, hybrid systems may integrate soft actuators with traditional motors or hydraulic units to balance flexibility and strength [138,139]. Such systems can offer a broader range of motion and more nuanced control, addressing a wider array of tasks and activities. The development of these hybrid actuator solutions reflects a growing emphasis on creating versatile and adaptable exoskeletons that can meet diverse user needs and operational requirements. In this section, studies developing active exoskeletons are reviewed, mainly focusing on the actuators and how they were selected for different type of work.

### 4.1. Conventional Actuators

Despite recent developments and advancements in smart actuators, conventional actuators are still widely used for active exoskeletons due to their proven reliability over a long period of time, availability, economical, high-power density, and customizability [140]. The most widespread and typical conventional actuators for exoskeletal solutions are electric motors, hydraulic actuators, and pneumatic actuators [135,141].

#### 4.1.1. Electric Actuators

Electric actuators are the most common types of actuators in active exoskeletal solutions, including direct current (DC) motors, servo motors (DC motors with sensors and control circuits), and stepper motors. This type of actuators offers precise control, a high power-to-weight ratio, and a compact size, making them ideal for applications requiring fine-tuned movements and dynamic response. Electric actuators are used in exoskeletons designed for medical rehabilitation, industrial assistance, and military applications that prioritize reliability and precision controls.

DC motors have been the most prevailing actuator type due to their affordability, high reliability, and high power-to-weight ratio [142]. Especially, brushless DC (BLDC) motors have become dominant in the DC motor market due to their efficiency and easy maintenance [143,144,145]. DC motors are better suited to lower limb supports because lower limb exoskeletons require high torque, significant load bearing, and high stability [146]. On the other hand, DC motors are not favorable for upper limb exoskeletons since upper limb exoskeletons require more sophisticated and dexterous movements [22]. Further, the excessive weights and sizes of DC motors, coupled with their drive system, could lead to fatigue and discomfort. Therefore, servo motors [147,148,149,150] and stepper motors [150,151] have been more widely used for upper limb exoskeletons than DC motors. These motors provide higher precision and dexterity, which, in turn, enables complex motion patterns and smooth operation, compared to DC motors, which are the key requirements of upper limb motions.

Electric actuators also possess several drawbacks that mainly arise from their additional components. While electric actuators themselves are generally compact, the necessary drive systems, such as gear and controllers, added to these actuators tend to be bulky and heavy. Furthermore, electric actuators require a power supply, typically in the form of batteries that limit the operational time of the exoskeleton. These added components increase the overall weight and size of the exoskeleton, which, in turn, potentially causes fatigue and/or discomfort of the wearer. DC motors have inherent problems associated with their dexterity. These motors may struggle with mimicking the full complexity of movements and the high degree of flexibility required, especially for upper limb exoskeletons. Servo and stepper motors that are known to provide more precise and smoother operations than DC motors require sophisticated control systems, sensors, and algorithms to manage movements.

#### 4.1.2. Hydraulic Actuators

Hydraulic actuators utilize pressurized fluid to generate mechanical motion, offering high force output. These actuators are commonly found in heavy-duty exoskeletons used in industries where lifting heavy loads and performing strenuous tasks are routine [152,153]. Hydraulic actuators have a superior power-to-weight ratio and provide great force compared to other actuators. However, this type of actuators tends to be heavy due to extra components, such as actuator cylinders, hoses, and auxiliary power supply units [154]. Furthermore, hydraulic actuators possess the potential risks of hydraulic fluid leakage, which could contaminate the wearer as well as other devices [155], and they are not highly compliant with safe human interaction [154]. Therefore, the use of hydraulic actuators is less common than the use of other actuator types in wearable robots unless a high amount of force is required [156]. To overcome these issues, an electro-hydraulic actuator (EHA), a hybrid system that combines both electric and hydraulic components, has been used for exoskeletons more recently. By taking advantage of both electric and hydraulic components, EHAs produce high force outputs while enabling precise and smooth controls and efficient energy use [142]. An electric motor is used to drive a hydraulic pump that controls hydraulic fluid to power the hydraulic actuator. Additional control systems are often embedded in EHAs to minimize the non-linear behavior of hydraulic systems [157].

#### 4.1.3. Pneumatic Actuators

Pneumatic actuators are driven by compressed air, providing lightweight and compliant behavior as compared to hydraulic actuators. In addition, they offer cleaner actuation systems while transmitting large forces and providing soft imposed movement and a reasonable power-to-weight ratio [155]. These actuators are often employed in exoskeletons designed for tasks requiring agility and adaptability, such as industrial assembly lines [153]. However, pneumatic actuators are known to lack the precision of electric actuators and require a bulky air supply system. The study of Xiang et al. [154] attempted to use a McKibben muscle in their exoskeleton filled with hydraulic fluids. They found that McKibben muscles with a hydraulic operating mode can provide minimal restrictions to fluid flow when joints move at high speed, while there was no difference in the performance as compared to the pneumatic operating mode. Moreover, these actuators lack fine controls, thereby limiting the exoskeleton’s tasks to less complex movements. Their reliance on compressed air leads to less accurate positioning and motion controls than electric actuators. In addition, this reliance may cause several issues, including limited and inconsistent force outputs and high noise levels. Therefore, the applications of exoskeletons with pneumatic actuators are limited to those requiring small assistive forces, such as rehabilitation therapy for fingers [158] and the wrist [159], and for providing supportive force only [160].

The performance of actuators in active exoskeletal solutions is influenced by various factors, including the power-to-weight ratio, efficiency, responsiveness, and durability. Actuators must provide sufficient power/torque output relative to their weight to ensure optimal performance without causing discomfort to the wearer. Actuators play a pivotal role in enabling the functionality and effectiveness of active exoskeletal solutions across diverse applications. By understanding the different types of actuators, their performance characteristics, and applications, researchers and engineers can contribute to the advancement of wearable technologies that enhance human capabilities. The key aspects of conventional actuators used in the recent development of exoskeletons, including weight, power, and torque/force, are shown in Table 2.

### 4.2. Non-Conventional Actuators

Soft robotics has enabled the use of shape memory alloys (SMA), silicone, and textile-based actuators in some exoskeletons such as upper limbs [165] and hands [166]. Soft actuators can align better to the joint movement and have a more natural feel to them. Most of these actuators are driven electrically, pneumatically, or using hydraulic (fluid) systems. Some of these actuators are discussed below.

#### 4.2.1. Shape Memory Alloy (SMA) Actuators

**Figure 4:**
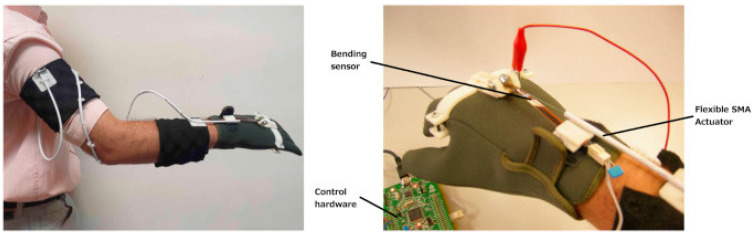
SMA wire-based actuator for wrist exoskeleton. (Reproduced from [168] with permission.).

SMAs are low cost, high strength-to-weight ratio actuators that are simple to implement in exoskeletons for rehabilitation purposes. An SMA actuator can be implemented in the shape of the wire that can, when heated, come back to its desired (memorized) shape after deformation. The output force of the actuator depends on the diameter of the alloy wire and the number of wires used. Another form in which these actuators can be implemented are springs. Springs have the advantage of providing much higher strain compared to the wire type SMAs. The output force depends on the number of springs used. In some cases, these basic SMA shapes are layered with other materials to form a composite structure [167]. Villoslada et al. [168] developed a 0.5 mm alloy wire based actuator to assist wrist movement, exerting a force of 35 N as shown in Figure 4.

While these actuators have the advantages mentioned earlier, they can suffer from hysteresis, which limits their frequency of operation, which can be undesirable for certain applications. Miniature fans have been used to improve the cooling rate of these actuators; however, not much success was reported, as most of the cooling was thought to be controlled by conduction rather than convection [169]. Alternatively, cooling fans were used to improve the cooling rate of an SMA actuator by a minimum of 70% in [170].

Some of the SMA-based actuators used in exoskeletons are compared in terms of their generated force and main element shape in Table 3.

#### 4.2.2. SMA-Based Soft Fabrics

SMA actuators have also been used to develop fabric muscles in [176], where spring bundles made of 0.5 mm wires were stitched in fabric to assist the wearer’s arms (elbow) and were able to generate a force of 100 N. Similarly, an alloy-wire-based wearable fabric actuator was developed by [177] to improve ankle plantar flexion. The actuator was able to create a movement/torque of 100 Ncm. A fan-integrated fabric muscle was developed by Park et al. [170] using a bundle of about 200 thin wire SMA springs to assist upper-arm load bearing and lifting. The muscle was reported to create a force more than 40 N and reduced the cooling time from 18.8 s to 5.6 s with the help of forced convection due to the fans. The overall weight was reported to be 30 g.

#### 4.2.3. Electroactive Polymer (EAP) Actuators

Dielectric elastomer actuators (DEA) are a form of EAP where a compliant dielectric layer is sandwiched between two compliant electrodes. When the voltage is applied across the electrodes, they attract each other, applying what is called Maxwell pressure, thereby compressing the layer between them. Due to this compression, the layer expands out increasing its cross-sectional area. The actuators can be stacked to generate more elongation and deformation. They can also be implemented in many other forms, such as rolled, tubular, and spring [178], and can be used to create artificial muscles. The Maxwell pressure depends on the magnitude of the applied signal and the material permittivity. Typically, signals at the order of 100 kV/mm are required to generate sufficient force and strain, which is unsafe for human use. Therefore, several materials have been used, such as silicone [179], acrylics [180], and polyurethane (PU) [181], to increase the permittivity and produce higher strains at relatively lower potentials.

Silicone has advantages like low creep, good thermophysical properties, and a long service life. However, typically, pure silicone has a relatively low dielectric constant (~3.0) and higher stiffness (low strain, usually <100%) compared to other dielectric materials used; therefore, efforts have been reported to increase the dielectric constant by mixing other higher dielectric materials such as titanium dioxide powder [182], polyaniline particles and silicon oil [183], and ferroelectric powder [184]. PU has better dielectric properties (~7.0) and can generate larger output force than silicone, but it can also be further enhanced by introducing graphene, carbon nanospheres (CNS), carbon nanotubes (CNT), and silicone elastomers [181].

**Figure 5:**
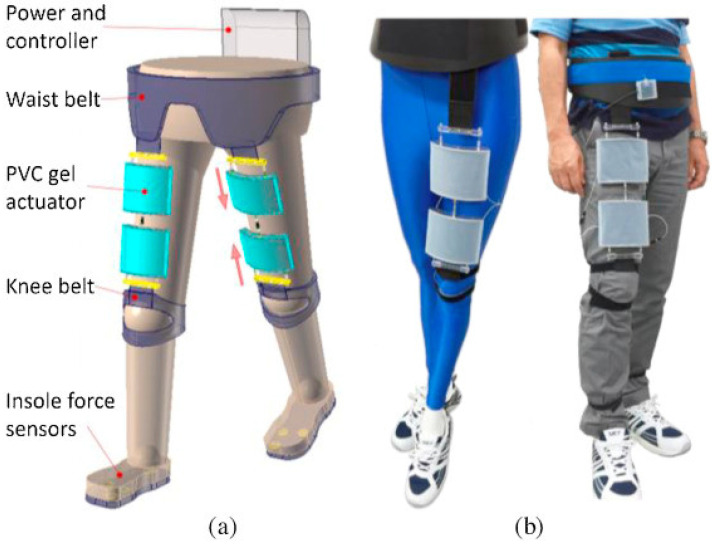
PVC gel based soft actuator for walking assistance: (**a**) CAD model (the red arrows show the creeping direction) and (**b**) manufactured actuator. (Reproduced under from [185] with permission).

Plasticized gels are another form of EAP that can produce actuation due to Maxwell pressure. A soft actuator using polyvinyl chloride (PVC) gel for walking assistance has been reported by Li and Hashimoto [185] (see Figure 5). Several multilayered actuators were used (10 layers each, with each layer 0.2 mm thick) to produce a displacement of 16 mm and a force of 94 N with a potential of 400 V. Baumgartner et al. developed a gelatin-based biogel that could produce a force of 14.7 N when pneumatically driven at a pressure of 102 kPa.

Thermo-responsive polymer threads constitute another class of muscles that can be used to actuate exoskeletons or body assistive systems. The polymer threads are twisted to increase the efficiency and power-to-weight ratio and are referred to as twisted and coiled polymers (TCP). TCPs have been produced using CNT yarn, polymer fishing lines, and conductive sewing threads. Out of these, Nylon 6,6 conductive sewing threads have been used to study the feasibility of these twisted threads to overcome hand spasticity [186].

#### 4.2.4. Actuator Limitations

Exoskeletal systems make use of different types of actuators, as presented in the above section. The choice of a particular actuator may depend on several aspects including the size, force and torque requirements, stiffness, control integration, movement accuracy, and body’s anatomical site. In addition to these technical aspects, an exoskeletal system’s appearance and acceptance by the user can also play a factor in the choice of actuator. The size of the actuator effects its overall dimensions and hence its appearance. Some limitation of the above-mentioned actuators are discussed below.

Electrically driven actuators constitute major part of the exoskeletons [71]; however, motors need to be controlled to match a certain speed–torque requirement of the joint or body site. Joints that require higher force or torque need bigger motors that add to the weight of the system if the motor is used to directly drive the joints. This worsens if series of motors are used for successive joints (e.g., along the arm). In this case, one motor needs to lift the other motor(s) in addition to lifting the arm. There are methods to avoid this by placing the motors far away from the joint removing the serial connection and driving the joints by links such as cables, chains, and, in some cases, rigid links. These links can, however, increase frictional losses in the system and increase its weight. The stiffness of these links also affects the output force or torque.

Pneumatic and hydraulic actuators are primarily adopted in lower limb exoskeletons [71] and can produce higher torques. Exoskeletal systems using hydraulic or pneumatic actuators are harder to implement, especially in portable exoskeletons, as they require pumps, compressors, and reservoirs to generate output. Moreover, regulators and valves can add to the cost and complexity of the whole system. Therefore, the usage of pneumatic and hydraulic actuators is limited to special applications where field mobility is not required. Different types of pneumatic artificial muscles (PAM) have also been used as actuators in exoskeletal systems [187]. They can be classified as soft robotics and have the advantages of flexibility, a higher force-to-mass ratio than electrical actuators, and being safer for use in human rehabilitation. However, they suffer from low bandwidth and non-linearities that require an accurate model to predict its dynamic behavior. These nonlinearities can also give rise to vibrations, which can be attenuated to some extent using complex control methodologies.

SMAs also suffer from some of the same limitations as PAM, as mentioned earlier (Section 4.2.1). Hysteresis and non-linearities can make the control regime of the actuators complex. The actuation cycle depends on how fast the material heats up, changes phase, and cools down. Single SMA actuators are usually fit for unidirectional motion only and pairs need to be used to generate bi-directional movement. Other issues include repeatability, reversibility, SMA degradation, especially at the material interface due to repetitive use, and materials shape memory effect [188].

EAP actuators are attractive options for exoskeletal actuators due to their light weight, smaller size biodegradability, and good mechanical properties. The advantages and disadvantages of the different types of EAPs depend on their construction and polymers used. However, the main drawbacks include higher driving voltages and actuation speeds. Dielectric and piezoelectric elastomeric polymers also suffer from low strain [189].

Despite the growing body of knowledge in the use of these actuators in exoskeletal systems for rehabilitation and occupational use, actual clinical tests and implementation are not extensive enough. User experience analysis is generally carried out through user surveys and questionnaires and, therefore, is subjective to the user experience instead of a general attribute of the exoskeletal system [190,191,192]. It is important to note here that most of the FDA (Food and Drug Authority, USA) approved medical exoskeletons for lower limbs use conventional motor actuators (e.g., Keeogo^TM^—knee orthosis from Keeogo, Quebec, Canada (neurological conditions), HAL^TM^—knee-hip orthosis, from Cyberdyne, Inc., Ibaraki, Japan, EksoNR^TM^—knee-hip, from EksoBIONICS, San Rafael, CA, USA, ExoAtlet^TM^ I and II, from ExoAtlet, Esch-sur-Alzette, Luxembourg, and ReWalk^TM^ from Argo Medical Technologies Ltd., Yokneam Illit, Israel).

## 5. Communication and Data Security in Active Exoskeletal Solutions

Most of the active exoskeletons communicate with external base stations through different technologies and the data security is of paramount importance. The communication with external base stations could be based on wired or wireless connection dependent upon the application. Wired communication technologies such as CAN (control area network) bus are employed by some researchers for the communication within the exoskeleton as well [193]. The advantage of CAN bus in exoskeletal applications is that each individual sensor or joint actuator can be considered as an independent device on the common bus. However, the disadvantage of CAN bus, from the security point of view, is that the CAN bus was developed in the 1980s for wired control area network protocol; it is a low-level protocol that does not have any inherent security protocols implemented at that level. Bozdal et al. have discussed the security challenges related to CAN bus in detail and the solutions for them [194]. All security measures need to be implemented by the application developer on the upper layers. Zhang et al. have developed a CAN-based inertial sensor network for a lower limb exoskeleton [195]. Zhou et al. have developed a lower limb exoskeleton robot using CAN bus for communication [196]. Another lower limb exoskeleton developed by Cao et al. used the CAN bus for communication [197]. There are other communication technologies used in exoskeletons, as shown in the Table 4. Zigbee is a widely used communication protocol with security. It uses IEEE 802.15.4 protocol and operates over 2.4 GHz, and it consumes less power.

There are many different kinds of security threats associated with wireless communication and there are various techniques to protect the data [198]. Data encryption is one of the key methods commonly used to protect exoskeleton data transmitted over wireless networks. The following encryption techniques are commonly used in wireless communications and Table 5 summarizes the main features of these encryption techniques.

- The Advanced Encryption Standard (AES) first introduced in 1997 [199], and developed by Rijmen and Joan in 2001 [200], is a widely utilized symmetric encryption method that is employed globally to secure data. The AES offers robust security using three differential sizes of keys.
- Rivest–Shamir–Adleman (RSA) is an asymmetric encryption method that is utilized to secure sensitive data, specifically for the purpose of exchanging secure keys (public and private keys).
- Transport Layer Security (TLS) is a protocol that guarantees confidentiality and security for communication between apps and users over the internet. Furthermore, this technology ensures complete security for the transmission of data.
- ChaCha20-Poly1305 is a stream cipher combined with a message authentication code (MAC) that provides authenticated encryption.

## 6. Active Exoskeletal Solutions

Active exoskeletons are sophisticated wearable devices engineered to enhance or restore human movement through the integration of advanced robotics and biomechanics. Unlike passive exoskeletons, which depend solely on mechanical support to aid the user, active exoskeletons are equipped with a combination of motors, sensors, and control systems that actively assist the wearer’s movements. These devices are designed to augment the physical capabilities of the user, providing additional strength, endurance, and support, thereby enabling the execution of tasks that would otherwise be challenging or unattainable [201,202,203].

The core functionality of active exoskeletons lies in their ability to seamlessly integrate with the human body’s natural movements. This integration is achieved through the use of sophisticated sensors that detect the user’s intentions and physical actions. Sensors such as accelerometers, gyroscopes, and force sensors capture real-time data about the user’s movements and the forces exerted on the exoskeleton [74]. These data are then processed by advanced control systems, which use algorithms and machine learning techniques to predict and respond to the user’s needs. The control system sends commands to the actuators—motors or hydraulic systems—that generate movement, providing the necessary assistance to the user’s joints and muscles.

### 6.1. Design Principles of Active Exoskeletons

The design of active exoskeletons involves balancing various factors to achieve optimal performance, comfort, and usability, such as biomechanical compatibility, weight distribution, adjustability, safety features, and user interface. The human joints and those of exoskeletons need to be biomechanically compatible to achieve human-centered kinematic solutions [204]. A study to collect the kinematic data of a patient transfer from a hospital bed to a surgery table was conducted by Tröster et al. [205]. Twelve optical motion capture cameras were used to track 41 markers attached to a human body and force plates and force-torque sensors were attached to the bed and floor to measure reaction forces. The exoskeleton design should complement the wearer’s biomechanics, aligning with natural joint movements and muscle activation patterns to minimize fatigue and discomfort. A similar setup with a motion capture system was used by Lee et al. [143] to analyze the biomechanics of human gait motion. This study, additionally, utilized electromyography (EMG) electrodes to monitor muscle activities as well as to cross-check kinematic data.

Active exoskeleton solutions tend to be heavier than their counterpart passive solutions due to their actuators, controllers, and power sources, with a typical weight range between 8 kg and 25 kg [206,207,208,209,210,211]. Therefore, efficient weight distribution across the exoskeleton is the key to prevent fatigue and strain on the wearer’s body without compromising mobility or agility. A lower-limb exoskeleton developed by Liu et al. [212] was simulated with four different designs for its optimum weight distribution by changing the placement of the motor and gearbox. This study indicates that the location of heavy components can alter kinematics, so the misplacement pronouncedly deteriorates its performance. One of the most famous active lower limb exoskeletons, BLEEX [213], has a weight distribution mimicking the weight distribution of the human lower limb to reduce interference. Another conventional approach is to place heavy components at the joints [214], thereby suppressing unintended effects.

There is no such unanimous kinematic model [215] whereby adjustability and customization is one of the critical design principles of active exoskeletons. Therefore, incorporating adjustable components and customizable designs allows the exoskeletons to accommodate different body sizes, user preferences, and tasks, eventually enhancing user comfort and performance. A pediatric gait assistive exoskeleton developed by Eguren et al. [211] consists of six joint control modules to suit various sizes of children with mobility limiting conditions. While braces need to be 3D printed for individuals, these modular components with actuators, microcontrollers, and torque and monitoring sensors can be attached as needed without disturbing the exoskeleton’s performance. For the assistance of upper limb motion, there was an effort to develop customizable upper-extremity active exoskeletons [216]. This proof-of-concept study proposed an exoskeleton design that can adjust the arm length to provide better fitments for multiple users sharing the exoskeleton when required.

With the advancement of technology for active exoskeletons, their field of application and the number of end users have rapidly expanded [217], which has led to increasing concerns over safety and user experiences. Wearable devices require a direct physical contact with their users. Therefore, they possess multiple potential safety risks, such as musculoskeletal injuries from improper use or fit and the malfunction of actuators [218], and skin irritation or chemical burns from battery leaks [14]. In recent years, a limited number of studies have been conducted to mitigate the potential safety issues of active exoskeletons. A couple of preventive systems were adopted by Li et al. [219] to improve the real-time interaction of the user with their gait training exoskeleton. An algorithm using visual feedback was proposed to estimate the user’s pose for necessary weighty supports, followed by the real-time adjustment of joint angles based on the EMG signals. These predictive devices improved individual’s user experience and reduced the risks associated with powered exoskeletons moving beyond the normal motion range of user’s joints.

### 6.2. Recent Trends in Active Exoskeleton Design

The design of active exoskeletons involves a sophisticated integration of various components, each playing a crucial role in the overall functionality and effectiveness of the device. An active exoskeleton typically comprises several key elements, each engineered to ensure optimal performance and user interaction.

#### 6.2.1. Frame Design

The frame of an active exoskeleton is a crucial component that provides structural support and facilitates the integration of other elements such as actuators, sensors, and control systems. Recent advancements in frame design reflect a trend towards increased customization, improved material performance, and innovative manufacturing techniques, all aimed at enhancing functionality, comfort, and adaptability.

Recent developments in materials science have significantly influenced frame design. Traditionally, frames were constructed from metals like aluminum or steel, which offered strength but often at the expense of weight [220]. Today, there is a shift towards using advanced composite materials, such as carbon fiber-reinforced polymers [221]. These materials are lightweight yet provide high strength-to-weight ratios, contributing to improved overall performance and comfort.

The trend towards increased customization and personalization of exoskeleton frames has been driven by the need to accommodate diverse body shapes and sizes. Recent advancements include the use of 3D printing and additive manufacturing technologies, which allow for the production of highly customized frame components tailored to the specific anatomy of individual users. This level of personalization improves comfort, fit, and effectiveness, making the exoskeleton more suited to varied applications and user needs [222,223].

Modular and adjustable frame designs are becoming more prevalent, allowing users to modify and adapt the exoskeleton according to their specific requirements [220,221,222,223,224]. Modular frames consist of interchangeable components that can be easily replaced or reconfigured, enabling users to adapt the exoskeleton for different tasks or conditions, thus enhancing its versatility. Adjustable components, such as telescoping limbs and adjustable joints, allow for real-time modifications to accommodate different postures and activities.

Recent trends also include the integration of soft robotics into frame design [225,226]. Soft robotic elements, such as flexible joints and deformable structures, are being incorporated to create exoskeletons that offer more natural and fluid movement. These soft robotic components work alongside traditional rigid frame elements to provide a more comfortable and adaptable user experience, helping to reduce the mechanical impact on the user’s body and improve overall ergonomics.

The focus on ergonomics and user comfort has intensified, with new frame designs incorporating features such as padded linings, adjustable harnesses, and ergonomic joint alignments [227]. These improvements aim to minimize discomfort and prevent pressure points during extended use. Advanced design techniques, including computational simulations and user feedback analysis, are used to optimize the ergonomic performance of the frame, ensuring alignment with the user’s natural body movements.

Minimizing the weight of the exoskeleton frame while maintaining structural integrity is a key trend [228,229]. Innovations in lightweight materials and design techniques, such as sandwich structures and optimized load distribution, are being employed to reduce the overall weight of the exoskeleton [230]. These advancements help improve energy efficiency and reduce the physical burden on the user, leading to enhanced mobility and reduced fatigue.

#### 6.2.2. Control System

Recent advancements in control systems for active exoskeletons are marked by significant improvements in computational power, adaptability, and real-time responsiveness. One major trend is the integration of advanced algorithms and artificial intelligence (AI) techniques to enhance the exoskeleton’s ability to interpret and respond to complex user movements [231,232]. Machine learning algorithms, including deep learning and reinforcement learning [232], enable the control systems to analyze large volumes of data from sensors and adapt to the user’s unique movement patterns. This sophisticated data processing allows for more intuitive and responsive assistance, as the exoskeleton can better predict and accommodate the user’s intentions and actions.

Another notable development is the incorporation of edge computing technologies within the control systems of active exoskeletons [233]. Edge computing enables the processing of sensor data locally on the device, reducing latency and improving the system’s responsiveness. By performing real-time computations on site rather than relying on remote servers, these control systems can provide more immediate feedback and adjustments to the actuators, enhancing the overall user experience. This advancement is crucial for applications requiring rapid and precise responses, such as dynamic and high-intensity movements.

Furthermore, there is a growing focus on the development of adaptive control strategies that allow exoskeletons to adjust their behavior based on varying conditions and user needs [126,234]. These adaptive control systems utilize feedback from multiple sensors to continuously adjust the level of assistance provided. Innovations in this area include the use of adaptive filters and model predictive control, which enable the exoskeleton to optimize its performance in real-time based on changing environments and user requirements. This adaptability improves the versatility of exoskeletons, making them more effective across different tasks and operational scenarios.

#### 6.2.3. Power Supply

Recent trends in power supply technology for active exoskeletons focus on enhancing energy efficiency, extending operational duration, and improving overall usability [235,236]. A significant advancement is the development of high-energy-density batteries, which provide greater energy storage in a lighter and more compact form. Innovations in battery technology, such as lithium-sulfur and solid-state batteries, are leading to improvements in energy density, safety, and longevity. These new battery types offer higher performance compared to traditional lithium-ion batteries, reducing the need for frequent recharging and increasing the practical use time of the exoskeleton.

Another trend is the exploration of energy harvesting technologies, which aim to supplement the power supply by capturing and converting ambient energy sources [42,237]. Technologies such as kinetic energy harvesters [238] and thermoelectric generators [239] are being integrated into exoskeleton designs to capture energy from the user’s movements or body heat. These energy harvesting methods can extend the operational life of the exoskeleton and reduce dependence on external power sources. For instance, kinetic energy harvesters can convert the motion of walking into electrical power, while thermoelectric generators utilize temperature gradients to generate electricity.

### 6.3. Active Exoskeletons

Active exoskeletons come in various types, each designed to address specific needs and applications. These types can be categorized based on their functionality, application area, and design features:

#### 6.3.1. Lower Limb Exoskeletons

Lower limb exoskeletons are designed to assist with or enhance the movement of the legs. These devices are often used in rehabilitation, mobility enhancement, and for assisting individuals with walking impairments [43,73,234,237]. They can provide support for walking, standing, and climbing stairs. In rehabilitation settings, they are used to help patients recover mobility after injuries or strokes. For industrial applications, these exoskeletons help reduce fatigue and strain from prolonged standing or walking.

These devices, designed to support or enhance leg movements, are seeing advancements in lightweight materials and advanced control algorithms. Recent trends include the development of exoskeletons with improved adaptability for uneven terrain and enhanced energy efficiency. Innovations in actuators and power systems are making these exoskeletons more comfortable and effective for both rehabilitation and industrial applications.

#### 6.3.2. Upper Limb Exoskeletons

Upper limb exoskeletons focus on assisting or augmenting the arms and shoulders [22,240,241]. These devices are commonly used in industrial settings to support workers who perform repetitive or strenuous tasks, such as lifting and carrying heavy objects. They can also be employed in rehabilitation to aid individuals recovering from shoulder or arm injuries, or to support people with conditions that impair upper-body movement.

Recent trends in upper-limb exoskeletons involve the increased miniaturization of actuators and sensors, leading to more compact and ergonomic designs. Enhanced machine learning algorithms are improving the precision of motion assistance, making these devices more effective for tasks requiring fine motor control. Additionally, there is a growing focus on integrating wearable biosensors to monitor and adapt to the user’s physical condition in real-time.

#### 6.3.3. Full Body Exoskeletons

Full body exoskeletons cover both the upper and lower limbs, providing comprehensive support and augmentation for the entire body [242,243,244]. These exoskeletons are used for a variety of applications including the military, rescue operations, and extensive physical rehabilitation. They are designed to enhance overall strength and endurance, allowing users to perform physically demanding tasks with reduced effort and increased stability.

Full body exoskeletons are benefiting from advancements in both material science and power management. Recent innovations include the use of advanced composites to reduce weight while maintaining strength, and the integration of hybrid power systems that combine high-energy-density batteries with energy harvesting technologies. These developments enhance the overall durability and functionality of full body exoskeletons, making them more practical for the military, rescue, and extensive rehabilitation applications.

#### 6.3.4. Medical and Rehabilitation Exoskeletons

Medical exoskeletons are specifically designed for rehabilitation purposes and to assist individuals with mobility impairments [58,76,226,234]. These devices often include features tailored to facilitate physical therapy, such as adjustable settings for varying levels of assistance, sensors for tracking progress, and systems for providing feedback. They are used in clinical settings to help patients regain mobility after surgeries, strokes, or spinal cord injuries.

Recent trends in medical exoskeletons include the use of adaptive control systems that can tailor assistance based on individual therapy needs. Improvements in real-time data processing and AI-driven algorithms are providing more personalized rehabilitation experiences. Additionally, there is an increasing emphasis on user-friendly designs that facilitate ease of use and integration into clinical settings.

#### 6.3.5. Industrial and Occupational Exoskeletons

Industrial exoskeletons are intended for use in workplace environments to enhance worker performance and reduce physical strain [37,201,245,246]. These exoskeletons are designed to support heavy lifting, reduce musculoskeletal injuries, and increase productivity. They are commonly used in sectors such as manufacturing, construction, and logistics, where workers are required to perform physically demanding tasks regularly.

In the industrial sector, recent trends focus on increasing the versatility and ease of use of exoskeletons. New designs incorporate modular and adjustable components to accommodate different tasks and body types. Advancements in wearable technology and IoT integration are allowing for real-time performance monitoring and data analytics, which help optimize worker safety and productivity.

#### 6.3.6. Military and Tactical Exoskeletons

Military exoskeletons are designed to enhance the physical capabilities of soldiers and other personnel in combat or tactical situations [59,245,247]. These exoskeletons often focus on improving strength, endurance, and load-carrying capacity, allowing users to carry heavy equipment and navigate challenging terrain more effectively. Features may include rugged designs, advanced control systems, and enhanced protection against environmental hazards.

For military applications, recent trends include the development of rugged, all-terrain exoskeletons with enhanced protection features and the integration of advanced navigation systems. Innovations in power systems, such as lightweight and high-capacity batteries, are extending operational endurance. There is also a focus on enhancing the stealth and mobility aspects of these exoskeletons to better support tactical operation.

#### 6.3.7. Assistive Exoskeletons for Daily Living

These exoskeletons are aimed at individuals who need assistance with everyday activities due to physical disabilities or aging-related conditions [248,249]. They provide support for walking, standing, and performing daily tasks, improving the quality of life for users by enabling greater independence and reducing the physical effort required for daily activities.

Recent advancements in assistive exoskeletons for daily living include the integration of flexible and adaptive design features that cater to varying levels of physical ability and activity levels. New technologies in sensor integration and user interfaces are making these devices more intuitive and responsive to the user’s needs, improving overall ease of use and functionality.

Table 6 summarizes the different types of active exoskeletons and their industry application, supported body part, and stage of development.

### 6.4. Challenges in Active Exoskeleton Development and Integration

Active exoskeletons, while presenting promising advancements in fields like rehabilitation, industrial work, and military operations, face a range of substantial challenges concerning performance, applicability, and integration. One of the primary performance challenges revolves around power consumption and battery life [208,255]. These systems rely on motors and actuators to assist human movement, and their significant power demands create a need for large batteries. However, these batteries are often limited in capacity, restricting the exoskeleton’s operational time, especially in high-demand environments such as industrial workplaces or prolonged military operations. The need for frequent recharging or swapping of batteries interrupts work and diminishes the overall utility of the devices. Additionally, balancing the exoskeleton’s weight and power output is a critical consideration. While heavier exoskeletons can support greater loads or provide more assistance, they may lead to increased user fatigue over time. Conversely, lightweight models may compromise on performance, providing inadequate support for intensive tasks. This trade-off between weight and functionality creates a design bottleneck that developers must carefully navigate.

Another performance-related issue is biomechanical alignment, which refers to the proper alignment of the exoskeleton’s joints with the user’s anatomical joints. Misalignment can create discomfort, reduce the efficiency of movement, and even pose risks of injury over extended use [256]. This challenge is compounded by the wide variability in human anatomy, meaning that one-size-fits-all designs are rarely effective. Misalignment may also disrupt the natural gait or motion of users, particularly in rehabilitation scenarios where patients need precise movement assistance. Furthermore, exoskeleton control systems are required to respond to the user’s movements in real time. Delays in response time or inaccuracies in interpreting the user’s intended actions can result in jerky or unnatural movement patterns, which reduce the system’s effectiveness and may lead to frustration or physical strain for the user. The complexity of fine-tuning these control systems to operate in sync with human motion adds an additional layer of difficulty in ensuring that the exoskeleton responds fluidly and intuitively to the user’s intentions.

From an applicability standpoint, exoskeleton designs are often highly task-specific, limiting their use across various industries or user groups [247]. Rehabilitation exoskeletons, for instance, are typically designed to assist with the restoration of basic movement patterns in patients with neuromuscular conditions, while industrial exoskeletons are geared toward enhancing physical strength and reducing the risk of injury during tasks such as lifting or repetitive manual labor. However, exoskeletons built for one purpose may not perform well in another, reducing their versatility and limiting widespread adoption across different sectors. Customization is another major challenge that influences the applicability of exoskeletons. Human bodies differ significantly in size, shape, and movement style, and exoskeletons often need to be customized to fit individual users. This customization process can be time-consuming and costly, particularly in large-scale deployments [257]. Mass production of a highly personalized product is inherently difficult, and the added complexity of customizing each unit leads to increased costs, both in terms of manufacturing and end-user affordability.

The learning curve associated with exoskeleton use presents additional applicability concerns [258]. Many exoskeletons require users to undergo extensive training to operate them effectively and safely. Without sufficient training, users may struggle to control the device properly, which can reduce efficiency, limit the benefits of the technology, or even cause harm. In industrial settings, where time is often critical, the need for prolonged training periods can be a deterrent to adoption. Similarly, in military applications, soldiers must be proficient in using exoskeletons in high-stress environments, adding another layer of complexity to their training. Durability is another key issue, particularly in sectors such as construction or military operations, where exoskeletons are subjected to extreme conditions, including exposure to dust, water, heat, and impacts. Ensuring that these systems can withstand harsh environments while maintaining optimal performance is a significant technical hurdle. Failure to achieve durability standards could result in frequent breakdowns or malfunctions, rendering the exoskeleton impractical for field use.

Integration challenges are perhaps the most technically demanding aspect of exoskeleton development. These systems rely on a complex network of sensors to detect the user’s movements, including motion, pressure, and force sensors. Integrating multiple sensors and ensuring that they work harmoniously without interference is a substantial challenge [259]. Accurate sensor data is crucial for the system to correctly interpret the user’s movements and provide appropriate assistance. Moreover, sensor fusion—the process of combining data from different sensors to create a unified understanding of the user’s actions—requires sophisticated algorithms that can process this information in real time. Delays or inaccuracies in data processing can lead to improper responses from the exoskeleton, reducing its effectiveness and potentially causing safety concerns.

Achieving seamless human–machine interaction is another major integration challenge. The exoskeleton must interpret the user’s movements and intentions accurately and adapt its actions accordingly [260]. This requires advanced control algorithms or machine learning systems that can not only respond to the user’s current movements but also predict their intended actions. For example, an industrial exoskeleton assisting with lifting tasks must be able to anticipate when the user is about to pick up a heavy object and adjust its support accordingly. Achieving this level of predictive control is complex and often requires continuous refinement through testing and user feedback. Additionally, the exoskeleton generates vast amounts of data from its sensors, which must be processed in real time to ensure smooth operation. The computational demands of processing these data while maintaining low latency are significant, and any delay in data processing could compromise the device’s performance.

Regulatory and safety concerns are also major barriers to the widespread integration of exoskeletons, particularly in healthcare and industrial settings [261,262]. These devices must meet stringent safety standards to ensure that they do not pose risks to users. For example, medical exoskeletons used in rehabilitation must undergo rigorous clinical testing to verify their effectiveness and safety before they can be approved for use in hospitals or rehabilitation centers. Similarly, industrial exoskeletons must comply with workplace safety regulations to ensure that they do not introduce new hazards. Meeting these regulatory requirements often involves lengthy testing and certification processes, which can slow down the development and deployment of new exoskeleton models.

Finally, the high cost of manufacturing, customizing, and maintaining active exoskeletons presents a significant economic challenge [263]. The materials and technology required to build exoskeletons—such as lightweight alloys, high-performance motors, and advanced sensors—are often expensive, driving up production costs. Customization further adds to these expenses, as exoskeletons often need to be tailored to individual users. Additionally, maintenance costs can be high, particularly in industrial environments where the exoskeletons may be exposed to wear and tear. These economic barriers limit the accessibility of exoskeletons, particularly for smaller companies or healthcare providers with limited budgets.

## 7. Conclusions

In this paper, we looked at the advancement of exoskeleton technologies, encompassing both passive and active systems, which represented a significant leap forward in enhancing human capabilities across various fields as well as providing a safe work environment for manual workers. This review has highlighted the critical role of actuators in shaping the functionality and performance of active exoskeletons. Conventional actuators—electric, hydraulic, and pneumatic—each offer distinct advantages and limitations. Electric actuators excel in precision and compactness, hydraulic actuators in force output, and pneumatic actuators in agility, though each presents unique challenges that impact its practical applications.

On the other hand, non-conventional actuators like shape memory alloys (SMAs) and electroactive polymers (EAPs) offer innovative solutions with the potential to revolutionize exoskeleton design. SMAs provide an effective means for rehabilitation with their unique shape memory properties, while EAPs present promising opportunities for developing soft, adaptable artificial muscles that can closely mimic natural movement.

With the increase of cyber security threats, the communication protocols used for exoskeleton data collection and control constantly under pressure to increase the security levels. At the same time, this provides a good opportunity for the researchers to implement additional security layers to the communication protocols used.

As the field of wearable robotics continues to evolve, understanding the comparative advantages and limitations of both conventional and non-conventional actuators is essential. This review aimed to serve as a valuable resource for guiding future research and development, contributing to the creation of more efficient, safe, and versatile exoskeletons. By leveraging the strengths of existing technologies and exploring new frontiers, researchers can advance the capabilities of exoskeletons and expand their applications, ultimately enhancing human performance, safety, and quality of life. The insights provided will contribute to creating more efficient, safe, and versatile exoskeletons, ultimately advancing human performance and mitigating the challenges associated with manual labor in various industrial settings.

## Figures and Tables

**Table 1:** Passive exoskeletons.

Name of the Exoskeleton	Passive Element	Supporting Areas	Country of Origin
Ekso EVO [15,16]	Spring Based Actuator	Shoulder	USA
Hilti Exo-001 [10,12]	Elastic Straps	Shoulder	USA
PULE (Passive Upper Limb Exoskeleton) [17]	Gas Springs	Shoulder	Taiwan
Levitate exoskeleton [18,19]	Springs	Shoulder	USA
Model-based Biomechanical Exoskeleton [20]	Springs	Shoulder	Germany
TasKi [21]	Springs	Shoulder	Japan
Skelex 360 [10,22]	Springs	Shoulder	The Netherlands
Pole harvesting support exoskeleton [23]	Springs	Shoulder	Malaysia
H-Vex [24,25]	Springs	Shoulder	Korea
ShoulderX by Suitx [26,27]	Springs	Shoulder	USA
Harpos MS [28,29]	Springs	Shoulder & Elbow	France
Static upper limb activity supporting exoskeleton [30]	Springs	Arm (Upper Limb)	Switzerland
Parallelogram type Exoskeleton [31]	Springs	Arm (Upper Limb)	Switzerland
Hero Wear Apex [15,16]	Elastic Straps	Back	USA
LiftSuit v2.0 (Auxivo AG) [32,33,34]	Spring (Fabric)	Lower Back	Switzerland
Three-layer Fabric Mechanism, Assistive Suit [35]	Elastic Fabric	Lower Back	Japan
IPWE (Industrial Passive Waist-assistant Exoskeleton) [35]	Elastic Straps	Lower Back	China
Laevo 2.0 [36,37,38]	Elastic Fabrics	Lower Back	The Netherlands
VT-Lowe’s Exoskeleton [39,40]	Carbon Fiber Legs	Lower Back	USA
Ez-UP [41]	Deformable and Non-Deformable Belts with Quadrilateral structured Elastic Fabric	Back and Upper Limbs	Japan
Lower limb energy harvesting and transmission exoskeleton (EHTE) [42]	Flat Spiral Springs	Lower Limbs	China
LegX by Suitx [43,44]	Springs	Knees	USA
Paexo Back from Ottobock [45]	Springs	Back	Germany

**Table 2:** Conventional actuators used in lower/upper limb exoskeletons.

Reference/Year	Actuator Type	Location/Purpose	Weight (g)	Power (W)	Torque/Force
Takamitsu et al. [153]/2009	Pneumatic	Upper limb/Elbow, shoulder, and waist support	5800 (entire exoskeleton)	N/A	Elbow & Shoulder 45 Nm Waist 90 Nm
Akdoğan and Adli [148]/2011	Servo motor	Lower limb/Rehabilitation	1600	570	1.15 Nm (stall)
Inose et al. [161]/2017	Pneumatic	Upper limb/back support	2900 (entire exoskeleton)	N/A	350 N @ 60 kPa
Zhang et al. [144]/2018	BLDC motor	Lower limb/Walking assistance	600	90	0.44 Nm
Pirjade et al. [162]/2020	DC motor	Lower limb/Hip and knee support	210	100	1 Nm (peak) 0.02 Nm (rated)
Bouteraa et al. [150]/2020	Servo motor	Upper limb/Elbow support for rehabilitation	152	36	2.4 Nm (stall)
Mahdavian et al. [147]/2020	Stepper motor	Upper limb/Arm support for rehabilitation	470	24	1.85 Nm (stall)
Lee et al. [145]/2021	BLDC motor	Lower limb/Ankle support	242	75	0.11 Nm
Sun et al. [152]/2021	Hydraulic	Lower limb/Walking assistance	2500	N/A	1700 N @ 18 MPa (with 4 actuators)
González-Mendoza et al. [149]/2022	Servo motor	Upper limb/Elbow support	153	93	1.68 Nm (rated) 8 Nm (stall) 20 N (axial)
	Servo motor	Upper limb/Wrist support	55	8	1.47 Nm (Stall)
Fang et al. [151]/2023	Stepper motor	Lower limb/Hip support for walking assistance	320	60	13 Nm (stall)
	Stepper motor	Lower limb/Knee and ankle support for walking assistance	320	55	6.5 Nm (stall)
Zhao et al. [163]/2023	Hydraulic	Lower limb/Knee support	1400 (without fluids)	N/A	160 N @ 60 kPa
Fan et al. [164]/2024	Hydraulic	Lower limb/Waking assistance with extra loads	4750 (actuator components)	N/A	237 N @ 2.5 MPa
Miškovic et al. [160]/2024	Pneumatic	Lower limb/Knee support	760 (without mechanical parts)	N/A	15.94 Nm @ 800 kPa

**Table 3:** SMA actuators used in exoskeletons.

Reference/Year	Main SMA Element	Location/Purpose	Weight	Force/Torque
Villoslada et al. Universidad Carlos III de Madrid [168]/2015	Wire (0.5 mm dia.)	Wrist	300 g	35 N
Hadi et al. (Univ. of Tehran) [171]/2018	Wire (0.25 mm dia.)	Hand rehabilitation	-	10 N each finger (40 N grasping)
Jeong et al. (Korea advanced institute of technology) [172]/2019	Spring (150 mm max. deformed length)	Wrist motion	151 g	1.32 Nm
Yang et al. (Northeastern University, China) [173]/2021	Spring (113 mm max. deformed length)	Hand rehabilitation	-	2.7 N
Zhang et al. (Dalian Univ. of Technology) [174]/2021	Wire (2 wires supported by bias spring)	Knee		40 N
Xie et al. (Univ. of Shanghai, China) [167]/2023	Springs with composite structure	Hand	120 g	6.4 N (max. for one finger)
Xie et al. Univ. of Shanghai, China) [175]/2023	Spring (4 springs)	Elbow	230 g wearable (877 g total)	100 N

**Table 4:** Wired and wireless communication technologies commonly used in exoskeletons.

Communications Technology	Transmission Rate (/Mbps)	Transmission Distance (/m)	Maximum Connections	Power Consumption (/mW)	Transmission Mode
ZigBee	0.2/0.04/0.25	10~300	216~264	3	Point-to-point
Infrared	1.521/4/16	10~100	2	10	Point-to-point
HomeRF	1/2	10~100	127	100	Point-to-multipoint
Bluetooth	1/2/3	10~100	7	100	Point-to-multipoint
RFID	0.212	10~100	2	~	Point-to-point
CAN bus	0.05/0.125/0.25/0.5/0.8/1.0	40~1000 (wired)	32~127	Varies	Point-to-multipoint

**Table 5:** Comparison of different encryption techniques.

Technique	Type	Key Length (/bits)	Strengths	Weaknesses
AES	Symmetric	128/192/256	High security, efficient in hardware/software	Requires secure key management
RSA	Asymmetric	1024~4096	High security for key exchange, widely supported	Slower for large data sets
ECC	Asymmetric	160~512	Similar security to RSA with shorter key lengths	Complex implementation, parameter sensitivity
TLS	Protocol	Varies based on the key type	End-to-end security, widely adopted	Requires proper configuration
ChaCha20-Poly1305	Symmetric with MAC	256	High performance, secure	Newer, less tested compared to AES

**Table 6:** Summary of active exoskeletons found in the literature.

Main Areas of the Supporting Area of the Body	Specific Area of Support	Name/Made	Power	Industry	Country of Origin	Year	Tasks That Can Be Supported
Upper Body	Upper Limb (Shoulder)	Armored 3DoF Shoulder Exoskeleton [59]	Active (Motors)	In research stage (military)	Spain	2020	Shoulder assistance
		H–Pulse [203]	Semi Passive (Springs and Active Support Control)	In research stage	Italy	2020	Overhead task Assistance
	Upper Limb (Elbow)	Power-Assist Exoskeleton [241]	Active (Pneumatic)	In research stage	China	2014	Power Assistance
	Upper Limb	No name, design and lab testing only [240]	Active (Motos and Gears)	In research stage	Japan	2018	Lifting, Posture Support
	Fingers	Double-Acting Soft Actuator (DASA) Based Robotic Glove [250]	Active (Pneumatic)	In research stage	China/Honk Kong	2023	Finger Extension/Flexion
Back	Lower Back	Dynamic Lifting aid Exoskeleton [201]	Active (Motors)	In research stage	Europe (Ireland, Netherlands, Italy)	2017	Lifting Assistance
Lower Body	Lower Limbs	MIT lower-body exoskeleton [58]	Active (Motor)	Military	USA	2009	Heavy Lifting, Load Carrying
		Lower Limb Exoskeleton [202]	Active (Motors)	In research stage	Japan	2019	Walking Assistance
		AWGAS (Assistive Wearable Gait Augment Suit) [50]	Active Passive (Pneumatic and Gel Muscles)	In research stage	Japan	2018	Gait/Walking Assistance, Postural Assistance, Bent (Knee) Task Assistance
	Knees	Endoskeleton Type Knee Joint Assist [51]	Active (Pneumatic)	In research stage	Japan	2021	Posture Support (Half Sitting and Crouching)
		Knee exoskeleton [251]	Active (Motors)	In research stage	Japan	2016	Lifting from Crouch Position
	Lower Limbs/Back	HULC [60,245]	Active (Hydraulic)	Military	USA	2009	Heavy Lifting, Load Carrying (Enhanced Load Capacity)
		CRAY X [58,252]	Active (Motors)	Manufacturing	Germany	2019	Lifting Heavy Loads
		Model A/Model Y [245]	Active (Motors)	Various industries that handle goods	Japan	2019	Heavy Lifting, Posture Support
	Lower Back/Top of Lower Limbs	No name, Design only [253]	Active (Motors)	In research stage	India	2022	Heavy Lifting
	Hip, Knee	Non-Exoskeletal Structure [55]	Active (Motors)	In research stage	Japan	2014	Walking Assistance, Power Assistance
Whole body	-	Raytheon/Sarcos exoskeleton [58]	Active (Motors)	Military	USA	2009	Heavy Lifting
	Separate modules for different areas	HAL [58,245,254]	Active (Motors)	Multipurpose	Japan	2019	Lifting, Posture Support
	-	Tokyo University of Agriculture and Technology—Exoskeleton [58]	Active (Motors)	Agriculture (support for elderly workers)	Japan	2009	Posture Support
	-	Guardian XO and Guardian XO MAX [242,245]	Active (Motors)	Manufacturing	USA	2019	Heavy Lifting

## Data Availability

Data are contained within the article.
